# French People’s positions on supervised injection facilities for drug users

**DOI:** 10.1186/s13011-020-00321-2

**Published:** 2020-10-14

**Authors:** Maria Teresa Munoz Sastre, Lonzozou Kpanake, Etienne Mullet

**Affiliations:** 1 CERPPS, Maison de la recherche, Federal University of Toulouse, 5 allées Antonio Machado, 31058 Toulouse cedex 9, France; 2 University of Québec – TELUQ, 5800, rue Saint-Denis, Bureau 1105, Montréal (Québec), Montréal, H2S 3L5 Canada; 3 Institute of Advanced Studies (EPHE), 17 bis, rue Quefes, Plaisance du Touch, 31830 Paris, France

**Keywords:** Supervised injection facilities, Personal positions, Amphetamines, Cocaine, Heroin, France

## Abstract

**Background:**

Supervised injection facilities have been set-up in many countries to curb the health risks associated with unsafe injection practices. These facilities have, however, been met with vocal opposition, notably in France. As harm reduction policies can only succeed to the extent that people agree with them, this study mapped French people’s opinions regarding the setting-up of these facilities.

**Method:**

A sample of 318 adults--among them health professionals--were presented with 48 vignettes depicting plans to create a supervised injection facility in their town. Each vignette contained three pieces of information: (a) the type of substance that would be injected in the facility (amphetamines only, amphetamines and cocaine only, or amphetamines, cocaine and heroin), (b) the type of staff who would be working in the facility (physicians and nurses, specially trained former drug users, specially trained current drug users, or trained volunteers recruited by the municipality), and (c) the staff members’ mission (to be present and observe only, technical counselling about safe injection, counselling about safe injection and hygiene, or counselling and encouragement to follow a detoxification program).

**Results:**

Through cluster analysis, three qualitatively different positions were found: Not very acceptable (20%), Depends on staff and mission (49%), and Always acceptable (31%). These positions were associated with demographic characteristics--namely gender, age and political orientation.

**Conclusion:**

French people’s positions regarding supervised injection facilities were extremely diverse. One type of facility would, however, be accepted by a large majority of people: supervised injection facilities run by health professionals whose mission would be, in addition to technical and hygienic counselling, to encourage patrons to enter detoxification or rehabilitation programs.

## Background

Unsafe injection practices are common among people who inject drugs [1]. Drug-related harms that are associated with these unsafe practices include overdose, endocarditis, and transmission of human immunodeficiency virus (HIV), hepatitis C virus (HCV) and a host of bacterial agents. The community is also at risk of contamination owing, for example, to unsafely discarded injection debris (e.g., needles and syringes) [2].

In some countries, supervised injection facilities have been set-up to curb risks associated with unsafe practices. The first one operated in Switzerland in 1986, as a public health initiative [3]. Following its success, it was replicated in many places in Switzerland: 14 facilities operated in 2019 [4]. The Netherlands followed the Swiss initiative, with 24 facilities operating in 2018 [5]. Other European countries then tested the idea. In Germany, for example, at least 24 facilities are now available in six federal states [6]. On a global scale, at least 78 legal facilities operated in 2018 [2].

Despite scientific evidence showing that supervised injection facilities reduce the number of overdose fatalities, reduce the prevalence of HIV and HCV among patrons, increase the number of individuals seeking treatment (e.g., detoxification services, opioid agonist products), and improve people’s quality of life in affected areas (e.g., by reducing instances of public injection and by reducing the quantity of drug-related litter in public parks), without being associated with an increase in criminality in the vicinity [7], opposition to them has been vocal [2, 8]. The main argument against their creation is morally grounded. Drug consumption is, according to these views, bad behavior; it must be punished and setting-up any kind of public structure in which drug use is tolerated sends the wrong message [8].

### Public views regarding supervised injection facilities

In the face of this unshakeable opposition, researchers have conducted public opinion surveys to determine whether the strong opposition to supervised injection facilities reflects the views of the majority of citizens or is a rearguard action by a self-righteous minority. These studies were conducted in Canada, where a facility has operated in Vancouver since 1990, in Australia, before and after the setting-up of a facility in Sydney in 2000, and in the USA, where no facility was operating at the time of the surveys.

A survey conducted in 2009 in Ontario [9] found that, among 968 participants, (a) 31% agreed that supervised “injection facilities should be made available to injection drug users to encourage safer drug injection,” (b) 48% agreed that they “should be made available if it can be shown that they reduce overdose deaths or infectious disease among users,” or, “if they can increase drug users’ contact with health and social workers,” and (c) 56% agreed that they “should be made available if it can be shown that they reduce neighborhood problems related to injection drug use.” These figures were higher than those found 6 years before in a similar study using the same material [10]. However, they were lower when it was the acceptability of supervised smoking facilities (e.g., for smoking crack cocaine) that was being assessed [11]. A survey conducted in 2011 in British Colombia found a higher level of support among the general public. A large majority (76%) of the 2000 respondents supported harm reduction strategies for people who inject drugs. Females, younger participants and more educated participants were more supportive than males, older and less educated participants [12]. However, support was lower in rural areas of the province [13].

A survey conducted in Sydney in 2000 found that 68% of the 724 respondents living in an area long affected by drug dealing and public drug consumption agreed that a supervised injection facility should be set-up in their area. Two years after it was created, public support increased to 78% (of 747 respondents). In addition, fewer respondents reported witnessing public drug use or publicly discarded drug-related litter. There was no change in the percentage of those that reported having been offered illicit substances in the street [14].

Last, a survey conducted in 2013 in the United States [15] found that 60% of the 899 respondents agreed that “supervised injection facilities for current intravenous drug users (i.e., legally sanctioned and medically supervised facilities to consume drugs) should be made available through federal funds if it can be shown that they reduce overdose deaths or infectious disease among users.” Agreement was higher among older participants and among those with liberal political ideologies and non-punitive views towards drug addicts. However, another survey, conducted in 2017 [16], found very different results. Only 29% (18% of Republicans and 39% of Democrats) of 1004 participants supported the legalization of supervised injection facilities. A subsequent study [17] found that arguments used to oppose legalization, such as “Safe consumption sites should be illegal because funding should be spent instead on opioid use treatment and recovery” and “Safe consumption sites should be illegal because use of heroin and other opioids is illegal,” were supported by 58 and 56% of the 1004 participants, respectively. Moreover, all the arguments commonly used to support supervised injection facilities were only approved by a minority in each case. Even an argument, based on the available evidence, that demonstrated these facilities were effective in reducing fatal overdoses failed to convince a large majority (66%).

### The present study

The present study was conducted in France. In 2017, about 2% of young French adults (18–34 years) were using amphetamines (or derivatives), and about 3% were using cocaine. In addition, 1% had experimented with heroin at least once in their life. The number of high-risk opioid users was estimated at 210000 [18]. Syringe exchange programs were introduced in France in 1990 [19]; about 12,000,000 syringes were distributed in 2017. Maintenance treatments were made legal in 1993 [20] and the number of clients receiving opioid-substitution treatment was about 180,000 in 2017. Each year, however, about 350 drug users die from overdose [18].

The French government has been contemplating setting-up supervised injection facilities for a long time without been able to reach a political consensus on the subject [8, 21]. Despite opposition from the French Academy of Medicine, the French Academy of Pharmacy, the French National Authority for Health (Haute Autorité de Santé) and the National Council of the Order of Doctors (Conseil National de l’Ordre des Médecins) [22], a facility was finally established in Paris in 2016 and, later in the same year, a second was set-up in Strasbourg. To encourage their acceptance by the local authorities, these two facilities were called *experimental facilities*, even though such facilities have existed in neighboring countries for more than 20 years. Their maximum duration of operation was set at 6 years.

Although some published data on people’s views about supervised injection facilities was available at the time of this initiative, the findings were, as in the case of the surveys conducted in the US, seemingly contradictory. A survey conducted in 2010 found that, among the 2300 participants, 72% disagreed that “to prevent risks to health … heroin users should be provided with special premises and equipment where they can inject their drugs” [23]. Another survey conducted the same year found opposite results: 66% agreed that medicalized premises where drug users can inject their drugs in satisfactory hygienic conditions in order to limit the transmission of the AIDS and Hepatitis viruses (similar to those that exist in neighboring countries) should be created in France [21]. A third survey conducted the same year also found that a majority (53%) agreed that “places financed by the local councils where drug users can freely come with their drugs in order to take them under medical supervision and hygienic conditions” should be opened in France [21]. However, a fourth survey conducted in 2012 using this same question found that a majority (55%) opposed the idea [21].

As people’s views seem to oscillate notably as a function of the way questions are framed [21], the present study examined people’s positions regarding the setting-up, in their city, of supervised injection facilities that varied as a function of determined characteristics: (a) the kind of substance that users would be allowed to inject (e.g., only amphetamines), (b) the type of supervision staff (e.g., trained volunteers recruited by the municipality), and (c) the type of counselling provided (e.g., technical counselling about injection and hygiene). Three qualitatively different positions were expected: principled opposition, principled acceptance, and a flexible position that would take into account the characteristics of the proposed facility [24].

Firstly, as variable percentages of participants in all the studies on people’s opinions reviewed above always expressed their complete disagreement with harm reduction strategies, we expected that a sizable percentage of participants would express principled opposition. That is, they would oppose the setting-up of a facility, irrespective of its characteristics. Secondly, as there was always a small percentage of participants that expressed strong support in most studies reported above, irrespective of the characteristics considered, we expected that another segment of our sample would express principled acceptance. That is, they would agree with the setting-up of the facility, irrespective of its characteristics.

Thirdly, a flexible position was also expected, which we thought would be the majority position. The existence of this position was inferred from the variations observed in the percentage of participants who agreed or disagreed as a function of the formulation of the items in the surveys reported above. Namely, we expected that (a) when the facility involved physicians and nurses as supervisors, when their mission was to provide more than technical advice, and when only amphetamine consumption was allowed, a majority of people would agree with the setting-up of the facility, and (b) when the facility did not involve physicians or nurses as supervisors, when the supervisors’ role was not well defined, and when heroin consumption was also allowed, a majority of people would disagree with the setting-up of the facility.

## Method

### Participants

The study was conducted in France, in the areas of Toulouse and Andorra. Toulouse is the fourth largest city in France, with approximately 1,400,000 inhabitants in its metropolitan area. The level of amphetamine and cocaine use in the region is slightly higher than that reported for the country as a whole [25]. If the experiment in Paris is considered successful, this city, because of its size, would be one of the cities where a supervised injection facility is likely to be established. Andorra is located south of the Toulouse region in the eastern Pyrenees and has about 40,000 inhabitants in its urban center.

The sample was a convenience sample of lay people and health professionals. The participants were 318 unpaid adult volunteers (including 9 physicians, 13 nurses and 12 psychologists). They were between the ages of 18 and 89 years (*M* = 35.52, *SD* = 15.31). The researchers contacted 600 people as they walked on city sidewalks during the day or, in the case of health professionals, at their offices. Of these, 53% participated. Table 1 shows the demographic characteristics of the sample. Some participants (*N* = 50) indicated their political orientation and some (*N* = 56) indicated whether or not they had personally experienced at least one type of addiction at any point in their lives (e.g., smoking tobacco, drinking alcohol or using illicit substances). The main reason given for not participating was a lack of time.

**Table 1:** Demographic Characteristics of the Sample. Composition of the Clusters

		Cluster			
Variable	Level	Not Very Acceptable	Depends	Always Acceptable	Total
Gender	Male	33 (25)^a^	60 (46)	37 (29)	130 (41)
	Female	30 (16)^a^	96 (51)	62 (33)	188 (59)
Age	18–25 Years	16 (19)	44 (53)^a^	23 (28)	83 (26)
	26–35 Years	18 (22)	43 (52)^b^	22 (26)	83 (26)
	36–49 Years	8 (11)^a^	38 (54)^c^	25 (35)	71 (22)
	50+ Years	21 (26)^a^	31 (38)^abc^	29 (36)	81 (26)
Location	Toulouse Area	54 (20)	127 (47)	87 (33)	268 (84)
	Andorra	9 (18)	29 (58)	12 (24)	50 (16)
Expertise	General Public	59 (21)^a^	144 (51)	81 (28)^a^	284 (89)
	Physician	3 (33)	3 (33)	3 (33)	9 (3)
	Other Health Prof.	1 (4)^a^	9 (36)	15 (60)^a^	25 (8)
Religious	Atheist	29 (16)	91 (48)	68 (36)	188 (59)
Involvement	Believer in God	22 (24)	46 (51)	22 (25)	90 (28)
	Regular Attendee	10 (26)	19 (50)	9 (24)	38 (12)
	Not Reported	2 (100)	0 (0)	0 (0)	2 (1)
Political Orientation	Left Wing	1 (6)	7 (41)	9 (53)	17 (5)
	Center	2 (11)	15 (79)	2 (10)	19 (6)
	Right Wing	6 (43)	7 (50)	1 (7)	14 (5)
	Not Reported	54 (20)	127 (47)	87 (33)	268 (84)
Addictions	None	5 (17)	15 (52)	9 (31)	29 (9)
	Some	5 (18)	11 (41)	11 (41)	27 (9)
	Not Reported	53 (20)	130 (50)	79 (30)	262 (82)
Total		63 (20)	156 (49)	99 (31)	318
Mean Acceptability		1.42	4.44	7.36	4.75

Note: Figures in parentheses are percentages calculated for each row. Figures with the same subscript are significantly different, *p* < .05 (Test of difference between two proportions)

### Material

The material was composed of 48 vignettes describing a realistic story and a response scale. The vignettes were created by orthogonally combining the levels of three factors: Type of drug consumed (amphetamines only, amphetamines and cocaine only, or amphetamines, cocaine and heroin) x Type of staff working in the facility (physicians and nurses, former drug users who have been specially trained for this task, current drug users who have been trained, or trained volunteers recruited by the municipality) x Type of mission (to be present and observe only, technical counselling about injection, counselling about injection and hygiene, or counselling and encouragement to follow a detoxification program). According to experts in toxicology, the three drugs have different levels of risks: heroin (2.74), cocaine (2.46), and amphetamines (2.11) [26].

The following is an example of a scenario in the vignettes: “The Council of the town in which you reside has decided to create a supervised injection facility where drug users will be able to inject their preferred drug under the supervision of trained staff. In this center, sterile syringes will be made available to users. The center will essentially welcome amphetamine users, but also cocaine users. It will be run by medical staff composed of physicians and nurses. These health professionals will be present during drug consumption and will observe each user’s behavior. They will give technical advice on how to minimize negative effects on the user’s veins when injecting. They will also give advice about hygiene and the risk of contagion. They will gather and secure all used syringes. To what extent would you accept the setting-up of this type of harm reduction center in your town?” The response scale was an 11-point scale with two anchors labeled “Not very acceptable at all” and “Always acceptable.”

### Procedure

Data collection took place from 2016 to 2018. Each person was tested individually, in a quiet room, usually in the participant’s home. Other participants were interviewed in a vacant classroom at the local university. The procedure followed Anderson’s [27] recommendations for this type of study (see also [28]). Participants took 25 to 45 min to complete the ratings. No participant voiced complaints about the number of vignettes or about the credibility of the proposed situations.

### Data analysis

A cluster analysis, using the K-means procedure [29], was applied first in order to detect qualitatively different patterns of ratings. As a solution with at least three clusters was expected, a three-cluster solution was the first one to be tested. Several solutions were subsequently also tested: a two-cluster one, a four-cluster one, and a five-cluster one. The three-cluster solution was retained because it was the one that produced the most meaningful findings.

An overall ANOVA was conducted with a design of Cluster × Drug × Staff × Mission, 3 × 3 × 4 × 4. Owing to the great number of comparisons, the significance threshold was set at .001. As the cluster effect and the three two-way interactions involving cluster were significant, three separate ANOVAs were conducted at the cluster level. An additional ANOVA was performed on the data from the subsample of 50 participants who indicated their political orientation.

## Results

**Fig. 1:**
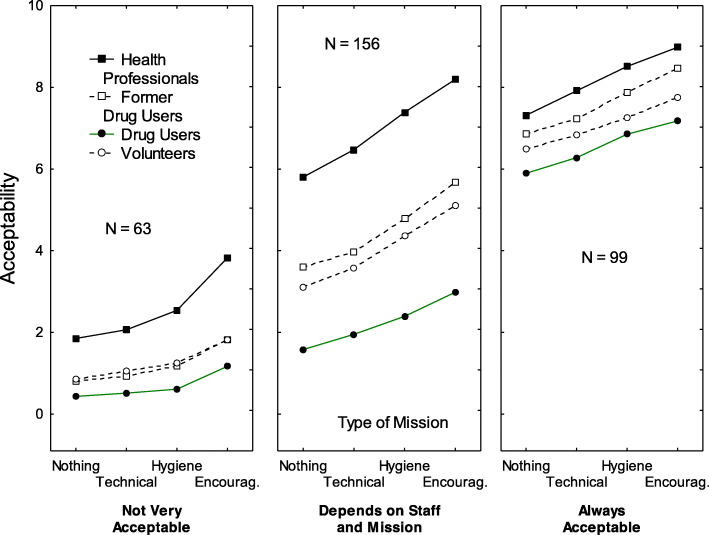
Pattern of ratings observed for the three clusters: *Not Very Acceptable* (left-hand panel), *Depends on Staff and Mission* (center panel), and *Always Acceptable* (right-hand panel). The y-axis corresponds to the acceptability judgments, the x-axis bears the four levels of the mission factor, and the four curves correspond to the four levels of the staff factor

The first cluster (*N* = 63, 20% of the sample) was the expected negative cluster. It was called *Not Very Acceptable* because, as can be observed in Fig. 1, most ratings were close to the unacceptable pole of the response scale (*M* = 1.42 out of 10, *SD* = 0.88). The only cases in which ratings were somewhat higher (*M* = 3.82, *SD =* 2.59) were when the vignette described having health professionals present in the facility whose mission was to encourage drug users to follow detoxification programs. As shown in Table 1, male participants and participants aged 50+ years were more often members of this cluster, compared with female participants and participants aged 36–49 years.

The second cluster (*N* = 156, 49%) was the expected pragmatic cluster. It was called *Depends on Staff and Mission* because these two factors were, as can be observed in Fig. 1 and in Table 2, by far the ones with the strongest impact on acceptability. Acceptability ratings were higher when (a) health professionals (*M* = 6.97, *SD* = 1.64) worked in the facility, rather than users (*M* = 2.23, *SD* = 1.72), former users (*M* = 4.51, *SD* = 1.82), or volunteers (*M* = 4.04, *SD* = 1.88), and (b) when the mission involved encouragement to follow detoxification programs (*M* = 5.49, *SD* = 1.40), rather than only advice related to hygiene (*M* = 4.73, *SD* = 1.11), injection (*M* = 4.00, *SD* = 1.13), or simple observation (*M* = 3.52, *SD* = 1.23). In addition, the effect of the center’s mission was stronger when staff in the facility were composed of health professionals (8.20–5.80 = 2.40), rather than users (2.98–1.58 = 1.40). As shown in Table 1, younger participants (18–49 years) were more often members of this cluster, compared with older participants (50+ years).

**Table 2:** Main Results of the ANOVAs Conducted at the Cluster Level. Results of the ANOVA with Political Orientation as a Between-Subject Factor

Cluster and Factor	*Df*	*MS*	*F*	*p*	η^2^_p_
Cluster Not Very Acceptable					
Type of Drug	2, 124	1.86	0.69	.50	.01
Type of Staff (S)	3, 186	492.48	32.91	.001	.35
Type of Mission (M)	3, 186	204.35	63.96	.001	.51
S x M	9, 558	11.91	9.07	.001	.13
Cluster Depends on Staff and Mission					
Type of Drug	2, 310	3.97	0.48	.62	.00
Type of Staff (S)	3, 465	7137.72	197.72	.001	.56
Type of Mission (M)	3, 465	1394.89	136.07	.001	.47
S x M	91,396	17.67	9.04	.001	.06
Cluster Always Acceptable					
Type of Drug	2, 196	2.32	0.43	.65	.00
Type of Staff	3, 294	579.57	28.94	.001	.23
Type of Mission	3, 294	477.15	67.15	.001	.41
Additional ANOVA					
Political Orientation	2, 47	1283.91	8.76	.001	.27
Type of Drug	2, 94	24.70	1.83	.17	.04
Type of Staff	3, 141	1153.34	33.56	.001	.42
Type of Mission	3, 141	334.59	39.10	.001	.45

**Fig. 2:**
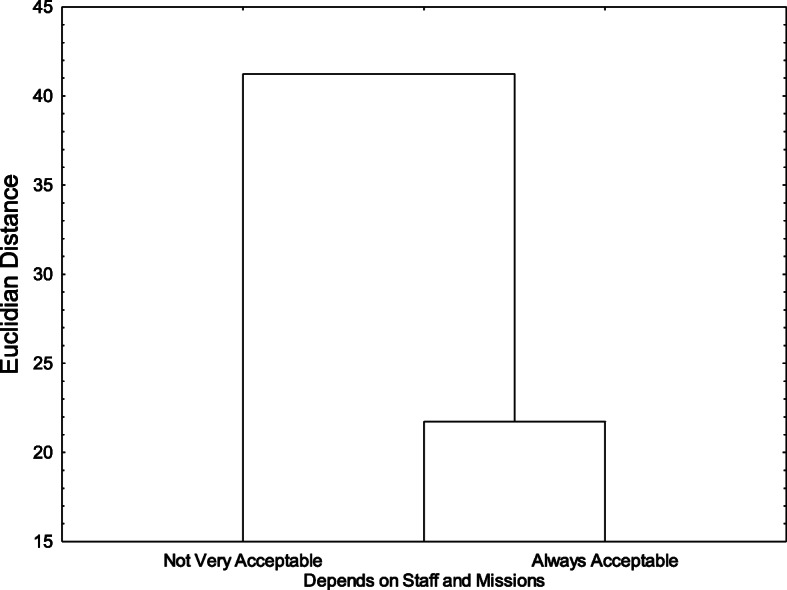
Euclidian Distances Between the Three Clusters

The third cluster (*N* = 99, 31% of the sample) was the expected positive cluster. It was called *Always Acceptable* because most ratings were close to the acceptable pole of the response scale (*M* = 7.36, *SD* = 1.03). There were no cases in which mean ratings were lower than the center of the acceptability scale. Figure 2 shows the Euclidian distances between the three clusters. Left-wing participants were more favorable to harm reduction centers in general (*M* = 6.08, *SD* = 1.76), compared with centrists (*M* = 4.43, *SD* = 1.32) or right-wing participants (*M* = 3.51, *SD* = 2.19).

## Discussion

The three expected positions were found. A minority of participants, above all males, older participants, and participants with a right-wing political orientation, expressed hostility to the setting-up of the proposed facility in their town. This result was consistent with the findings of previous studies showing that (a) young Canadian females were more favorable to such facilities that older Canadian males [12], and (b) participants with liberal political ideologies were more favorable than conservative participants [15]. Interestingly, however, in the unique vignette where health professionals were involved whose mission was to encourage drug users to enter detoxification programs, the level of hostility expressed was not as absolute as in other cases. This means that, even among those who oppose the setting-up of supervised injection facilities, some may change their mind if the medical and rehabilitative character of the project was emphasized.

Another minority of participants expressed a position of acceptance to the setting-up of a supervised injection facility in their town. Participants with a left-wing political orientation and health professionals (except physicians) expressed this position more often than other participants. This result was consistent with findings of previous studies mentioned above [15] and with the idea that nurses and psychologists may view drug users as people to be cared for rather than prosecuted. Although the opinions of these participants were generally favorable, they were less so when it was proposed that the facility be run by drug users themselves.

Finally, half of the participants, especially younger ones and those with a centrist political orientation, expressed nuanced views. These participants were always favorable to the setting-up of the facility when health professionals were involved in the project, above all if the mission of these professionals was to encourage users to enter detoxification programs. Further, these participants were always hostile to the proposal when only experienced drug users were involved in the project, even if these individuals were expected to encourage users to enter detoxification programs. In both of the other cases, that is, when former users or trained volunteers were involved in the project, these participants’ views were slightly positive or slightly negative, depending on the mission assigned to these individuals.

Overall, the factor with the greatest impact on acceptability was the type of staff involved in the project. This finding suggests that introducing supervised injection facilities as medical centers would enhance people’s willingness to agree with their setting-up in their town, even among people who would normally express negative principled positions. On the contrary, introducing supervised injection facilities as welcoming locations, where drug users can meet with one another and exchange experiences without any professional supervision, would completely abate people’s willingness to agree with their setting-up, even among people who would normally express positive principled positions. Political opponents to the setting-up of these facilities tend to use terms such as “shooting-up rooms” (in French “salles de shoot”) to convey the idea that these centers are pleasant locations where individuals indulge in the consumption of illicit substances with impunity. In one sense, they are very astute in presenting this idea because it is probably the best way to rally new opponents to any harm reduction project. In France, the establishment of the two experimental facilities mentioned above was made possible only once their final locations were, for one, at a hospital in the north of Paris and, the second, on the periphery of Strasbourg [8].

Overall, the second most important factor was the type of mission assigned to staff members. This finding suggests that introducing supervised injection facilities as a gateway to rehabilitation centers and detoxification programs would enhance people’s willingness to agree with their setting-up in their town, even among particularly reluctant people. On the contrary, introducing them as welcoming locations, where drug users can learn how to best avoid the negative consequences of their addictions, would largely abate people’s willingness to agree with their setting-up, even among particularly favorable people.

Interestingly, the type of drug allowed had virtually no effect on people’s opinions. However, this does not mean that people were blind to this factor. On average, facilities where only amphetamines were used were considered slightly more acceptable than those in which heroin was also used, but the difference was small. One way of interpreting this result is to consider that the general public is probably not very aware of the different risks associated with injecting the three substances mentioned in this study, hence the absence of a strong observed effect for the type of drug factor. Alternatively, it is possible that, from many people’s point of view, all three behaviors are equally bad. From a moral point of view, therefore, there is no difference in the acceptability of any of them. If this second interpretation were true, insisting that only moderately toxic substances are allowed would not be effective in convincing reluctant people to accept the facility.

### Limitations

One limitation of this study was that the sample was a convenience sample of lay people and health professionals. However, this study was not epidemiological in character. Rather, its aim was simply to map, as precisely as possible, people’s positions in regards to the acceptability of the setting-up of a supervised injection facility in their town, as a function of three pre-determined characteristics of the facility. Its aim was not to determine the exact percentages of individuals in the whole population that hold these positions. Future studies should, using a shortened version of our material, analyze the positions of fully representative samples of French adults and compare them with the positions of people from different parts of France.

A second limitation of the study was that, in each case, the decision to create an injection facility was taken by a political authority. This may have influenced the participants’ responses [30]. Future studies should assess the impact of this factor by comparing people’s responses in cases where the decision was supported by municipal authorities, regional authorities, the State, or was simply a local initiative from a charitable association.

A third limitation was that only three types of substances were considered, hence the difficulty in interpreting the lack of a strong effect for this factor. Future studies should assess further the impact of the kind of substance on people’s levels of acceptability by broadening this factor and including, in addition to substances that are injected, substances that can be inhaled, such as cannabis, where the effect on health is certainly perceived as less severe by the public.

## Conclusion

In 2010, when the then French Minister of Health introduced the idea of creating a supervised injection facility in Paris [31, 32], she (a) carefully avoided using the common French expression – “salles de shoot” – that would have been likely to deter other members of her government from supporting the project, and she (b) used two expressions that would logically have convinced them to show at least some measure of support: she presented supervised injection facilities as a first step to stopping illicit drug consumption – in French, a “premier palier pour arrêter la consommation de drogue” – and as the best way to move towards an abstinence policy – “la meilleure façon de passer à une politique de sevrage.” Despite her assertions being ethically sound [33], supported by current scientific evidence [2, 7], by the French National Institute for Medical Research [34], by positive reports from neighboring countries (e.g., Switzerland, Germany, and the Netherlands), and that they would have been in line with recommendations from the European Union [35], her project was vocally opposed by fellow party members.

In brief, even if a health minister’s proposal is supported by long-term experience abroad and scientific evidence, it has no chance of being adopted if political colleagues fear that the project will trigger strong public opposition, and may even lead to the government’s collapse. The present study shows that, under certain conditions, a majority of people would not oppose the setting-up of an injection facility. These conditions are that the facility is run by health professionals whose mission would be, in addition to technical and hygienic counselling, to encourage patrons to enter detoxification or rehabilitation programs. These conditions are consistent with the terms chosen by the Minister of Health 10 years ago when she presented her project.

Informing policymakers about citizens’ views on injection facilities remains a crucial objective because, in many places, largely unfounded fears have clashed with genuine public health considerations. In addition to the assessment of the effects of drug, staff and mission on people’s judgments, an assessment of the effect of additional factors would be welcome. Some of these factors are (a) the exact location of the injection facility, in particular the distance between the proposed facility and the participant’s home, and whether the facility would be close to the city center and/or a hospital, (b) the durability of the facility (temporary or not), (c) the presence and action of police in the neighborhood, and (d) whether people living in the neighborhood would be compensated for any negative consequences. A comparison of the positions of different groups of people would certainly be informative: lay people and health professionals, as in the present study, but also individuals running small businesses that may be affected by the proximity of an injection facility, members of the local police, people working in emergency services (ambulances), and potential patrons [36]. In other words, it is necessary to consider not only factors related to the health benefits that drug users can expect from the setting-up of a facility, but also factors related to the level of nuisance that people living nearby might expect, and to contrast these different points of view.

## Data Availability

All data collected is available and can be accessed by contacting the corresponding author.

## Abbreviations

HIV: Human immunodeficiency virus
HCV: Hepatitis C virus
SD: Standard deviation
ANOVA: Analysis of variance
M: Mean
